# Occupational Exposure to Solar UV Radiation of a Group of Fishermen Working in the Italian North Adriatic Sea

**DOI:** 10.3390/ijerph16163001

**Published:** 2019-08-20

**Authors:** Alberto Modenese, Francesco Pio Ruggieri, Fabio Bisegna, Massimo Borra, Chiara Burattini, Elena Della Vecchia, Carlo Grandi, Anna Grasso, Luca Gugliermetti, Marco Manini, Andrea Militello, Fabriziomaria Gobba

**Affiliations:** 1 Department of Biomedical, Metabolic and Neural Sciences, University of Modena & Reggio Emilia, 41125 Modena, Italy; 2 Department of Astronautical, Electrical and Energy Engineering, University of Rome “Sapienza”, 00185 Rome, Italy; 3 Department of Occupational and Environmental Medicine, Epidemiology and Hygiene, Italian Workers’ Compensation Authority (INAIL-DiMEILA), Monte Porzio Catone, 00078 Rome, Italy; 4 Studio Manini, Workers’ Health Surveillance Service, 47841 Cattolica, Italy

**Keywords:** solar radiation, ultraviolet radiation, occupational exposure, fishermen, risk evaluation, skin cancer prevention, workers health, exposure assessment, personal dosimetry, occupational safety and health

## Abstract

Occupational solar radiation exposure is a relevant heath risk in the fishing sector. Our aim was to provide a detailed evaluation of individual UV exposure in three different fishing activities in Italy, with personal UV dosimeters and a simple formula to calculate the fraction of ambient erythemal UV dose received by the workers. The potential individual UV exposure of the fishermen was between 65 and 542 Joules/m^2^. The percentages of the ambient exposure were estimated between 2.5% and 65.3%. Workers’ UV exposure was mainly influenced by the characteristics of the work activity, the postures adopted, and the type of boats. Overall, our data showed that 43% of the daily measurements could result largely above the occupational limits of 1–1.3 standard erythemal dose (i.e., 100 Joules/m^2^) per day, in case of exposure of uncovered skin areas. Measurements of individual UV exposure are important not only to assess the risk but also to increase workers’ perception and stimulate the adoption of preventive measures to reduce solar UV risk. Furthermore, the simple method proposed, linking ambient erythemal UV dose to the workers’ exposure, can be a promising tool for a reliable assessment of the UV risk, as time series of environmental UV dose are widely available.

## 1. Introduction

Solar ultraviolet (UV) radiation exposure is a relevant occupational risk factor for outdoor workers (OWs), and it may determine severe health consequences: UV rays are the leading cause of skin cancers, as excessive exposure to UV radiation has been linked to both melanoma and nonmelanoma skin cancers (NMSC) [1,2,3,4,5]. Biological evidence indicates that DNA damaged by UV exposure results in increasing rates of melanoma, basal cell carcinoma (BCC), and squamous cell carcinoma (SCC) [2,6]. Furthermore, UV exposure is responsible also for other skin diseases, such as actinic keratosis and photo-aging [1,6,7], and it is involved in the pathogenesis of several ocular disorders, such as cataract, pterygium, and possibly macular degeneration [6,7,8,9,10,11]. It has to be noted that not only high energetic UV-B rays are able to induce acute and chronic adverse effects, but also longer UV-A wavelengths, which are largely represented within the solar radiation reaching the Earth’s surface, have a great ability to photochemically interact with biological tissues, generating oxidative damages, and therefore playing a major role in photo-immunosuppression and cellular senescence, also after short-term exposures [2,12,13].

For the majority of these adverse health effects, scientific studies show a strong association with cumulative UV exposure, typical of outdoor work, even if to date, there is still little knowledge on the relations between UV doses received and the increasing risk of long-term, adverse eye and skin effects in humans [6,7,8,9,10,11,14,15]. Among the main reasons, there are the issues in reconstructing the cumulative dose received during several years of exposure: Many indirect models have been proposed in scientific literature with this purpose, but their application in epidemiologic research has some practical difficulties (e.g., when recollecting a reliable exposure history, due to subjective investigation and lack of objective individual UV exposure data) [14,15,16,17].

On the other hand, many studies investigated short-term outdoor workers’ UV exposure at different latitudes: Most of these studies are usually conducted in occupational sectors like agriculture and construction [7,18,19,20,21,22], while few studies have investigated solar UV risk in the fisheries sector, at least in Italy [7,15,23,24,25]. Fishing is a popular activity in the Mediterranean region, and fishermen spend many days outside on boats with very few or no possibilities of shielding their bodies. Furthermore, UV irradiance, directly depending on the incoming solar energy, is largely increased by the water reflectance properties (albedo phenomenon) [15,16,26].

For these reasons, the aim of this research was to perform a targeted assessment of individual UV exposure with personal electronic dosimeters in a group of fishermen in the North Italian Mediterranean area, investigating the characteristics of the exposure in different fishing activities and comparing individual exposure with environmental cloud-modified erythemal UV doses.

## 2. Materials and Methods

We performed an on-field UV measurement campaign with personal electronic UV dosimeters during two spring days (15–16 May 2018) in a group of fishermen working on three different boats for a fishing company of the Northwest Adriatic Sea (43.9°N, 12.7°E).

**Figure 1:**
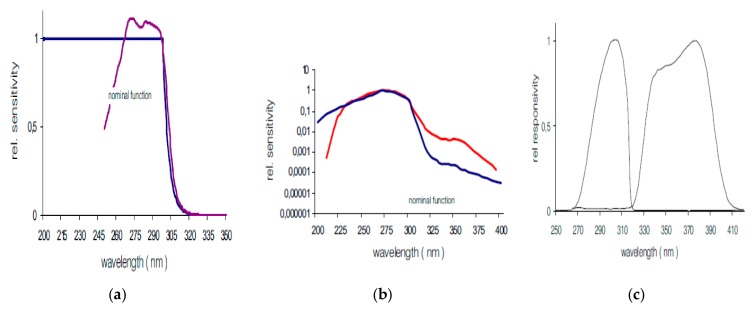
Relative sensitivity of the different types of electronic dosimeters used for the individual UV measurement campaign in fishermen in comparison with the relative weighting function (curves in blue): (**a**) erythemally weighted; (**b**) International Commission on Non-Ionizing Radiation Protection weighted; (**c**) unweighted (with a specific response, which is a function of the wavelengths).

We used nine electronic dosimeters Gigahertz-Optik X2000 series with different spectral weightings [6,27,28,29], i.e., erythemally weighted (Figure 1a, four dosimeters of this type were used), International Commission on Non-Ionizing Radiation Protection (ICNIRP) weighted (Figure 1b, three dosimeters of this type were used), and unweighted (Figure 1c, two dosimeters of this type were used; NB: It has to be noted that both these sensors have their specific wavelength response function), and two electronic dosimeters Gigahertz-Optik X2012 series, erythemally weighted. The details of the characteristics of the dosimeters are also shown in Table 1.

For a better comparison of the data provided by the different dosimeters, we tested them before the measurement campaign in standard conditions, i.e., on the horizontal plane, directly exposed to the sun in the same place, with a cloud-free sky. Then, we compared the irradiance values measured by the dosimeters with the effective erythemal irradiance measured on the same plane with a Gigahertz-Optik BTS2048-UV-S spectroradiometer, which was adopted as reference. Accordingly, it was possible to determine the different multiplicative coefficients for each of the instruments, to normalize the acquired data with respect to those measured by the spectroradiometer [30].

**Figure 2:**
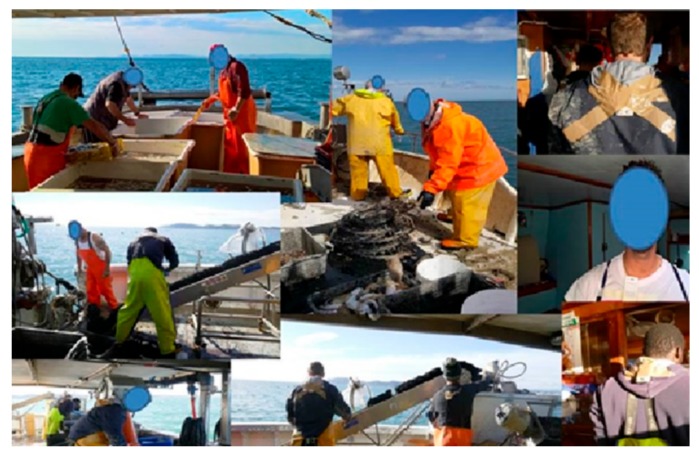
Placement of the personal UV dosimeters in the group of fishermen in different boats.

We applied the dosimeters on the back and/or on the chest or the nape of the fishermen: The choice of the body sites was made according to the need of not interfering with the fishermen’s job, monitoring the most exposed areas in stable and reproducible conditions, and also trying to protect as much as possible the surface of the sensors from water splashes (Figure 2).

**Figure 3:**
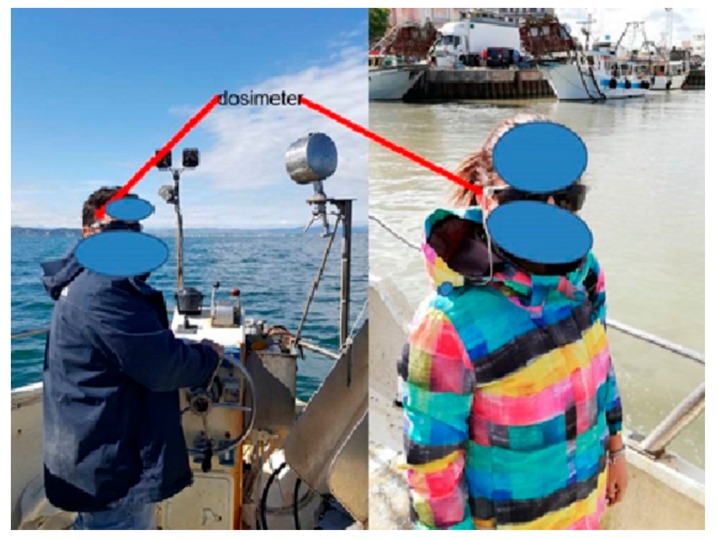
Personal UV dosimeters on the sunglasses of the research investigators on the boats.

Furthermore, we also used an ocular UV dosimeter to be worn on a pair of sunglasses (Figure 3): In this case, the dosimeter was worn by two volunteers of the research team on board of the boats (M.B. and A.G.), as the device was not suitable for use during all the phases of the fishing work.

We monitored two standard workdays of the fishermen from 07:00 to 14:00, and we registered on a diary the meteorological conditions, which were partly cloudy during the entire period. According to the Tropospheric Emission Monitoring Internet Service (www.temis.nl), the cloud-modified erythemal UV dose measured in Venice (less than 200 km north compared to the place of the measurement campaign) on 15th May was 2.6 kJ/m^2^, while on 16th May, it was 3.6 kJ/m^2^. Based on a duration of solar insolation in two days of 14 h and 30 min, we estimated the fraction of the cloud-modified erythemal UV dose of interest for the fishermen during their working activities, considering that approximately 30% of the total amount of UVR (ultraviolet radiation) reaches the Earth’ surface between 11:00 and 13:00, 45% in the period between 09:00 and 11:00 plus the period between 13:00 and 15:00, and finally the remaining 25% of environmental UVR reaches the ground in the period between 06:00 and 09:00 plus the period between 15:00 and 20:30 [31].

We performed the personal UV measurements on fishermen engaged in three types of fishing activities on different boats: mussels fishing, sea snails and cuttlefish fishing, and trawling. For each activity, a full description of the work phases was collected and summarized as follows.

(a) Mussels fishing: medium size boat, partially shielded from sunlight. The fishing phases were preparation of the net bags for the mussels’ grafting, sowing phase, mussel collection, choice of the mussels collected, and preparation of the net bags with the caught fish to be sold. It has to be noted that the sowing and collection phases were performed in direct sunlight.
(b) Sea snails and cuttlefish fishing: small boat, with no possibility of shielding from sunlight. The fishing phases were throwing down of the net bags and net baskets in the sea, collection of the caught fish with a pulley, and separation of the sea snails and cuttlefish from the other fishes.
(c) Trawling: medium size boat, partially shielded from sunlight. The fishing phases were casting of the nets, navigation phase to pull the nets, pulling up of the nets, choice of the caught fish, and preparation of the net bags to be sold. Exposure to direct sunlight was mainly during the net casting and pulling up phases.

This study was conducted within a campaign of monitoring of the occupational risk and information and training of the workers in full accordance with Italian national regulations on occupational health and safety, and with the principles of the Declaration of Helsinki. Data used in this study represent external exposure data, which were made freely available for the fishing company involved to be possibly included among the occupational risk assessment at the workplace, which is a mandatory requirement for all Italian companies. No evaluations of individual biological exposure and no estimates of any health outcome have been investigated in this study. The results of the targeted exposure measurements are representative of a standard working activity in a standard working day, not directly linked to specific persons, and these data cannot be extrapolated from an anonymous collective interpretation. Accordingly, approval from an ethic committee for this industrial hygiene study was not needed nor sought. 

Complete information regarding the study project was given to all participants, and subjects were informed that the presentation of the results in a scientific paper was on a voluntary basis, and that they were free to ask for the cancellation of their data at any time. An informed consent was collected. Nobody refused to participate or withdrew during the study.

## 3. Results

A total of 7 male fishermen, mean age 38.1 years, working on three different fishing boats were monitored for their occupational solar UV exposure with personal UV dosimeters.

Three fishermen on the first boat (fisherman—FM 1, FM 2, and FM 3) engaged in mussel fishing activity were respectively monitored with 3 devices placed on their back, for a total of 397′ between 07:00 and 13:37 on the first day, and for a total of 210′ between 07:00 and 10:3 during the second day.

Considering the sea snail and cuttlefish fishing activity, FM 4 and FM 5 on the second boat were monitored with 3 dosimeters placed on the back and on the nape for FM4, and on the back for FM5, for a total of 178′ between 08:43 and 11:41 during the first day, and for a total of 180′ between 07:00 and 10:00 during the second day.

Finally, in the trawling activity, FM6 and FM7 were monitored with 3 dosimeters respectively placed on the back and on the chest of FM6 and on the back of FM7 for 261′ on the first day between 08:15 and 12:36, and for a total of 300′ min between 07:10 and 12:10 during the second day.

The results of the individual solar UV exposure measurements for the different body sites of the 7 fishermen are shown in Table 2 for the first day of measurements (15th May 2018) and in Table 3 for the second day of measurements (16th May 2018). We also report the fraction of the total amount of environmental UV exposure received by the workers during their working time, calculated according to the procedure described in the Materials and Methods section.

Personal UV exposure at the back of the fishermen during the first day of the campaign ranged from 65 to 213 J/m² for the mussel fishing activity, from 288 to 542 J/m^2^ for the sea snail and cuttlefish fishing, and from 32 to 84 J/m² for the trawling activity (Table 2). On the second day of the measurement campaign, fishermen engaged in mussel fishing experienced a back UV exposure varying from a minimum of 29 J/m² to 71 J/m², for workers involved in the sea snail and cuttlefish fishing activity, we registered a UV exposure ranging from 84 to 284 J/m², and finally, for fishermen engaged in the trawling activity, the exposure was between 129 and 151 J/m² (Table 3).

The estimated proportion of environmental erythemal UV dose received on the back of the fishermen varied according to a percentage of individual vs. ambient UV exposure, ranging from 2.5% for fisherman FM 7, involved in the trawling fishing, to 65.3% for worker FM 4, engaged in sea snail and cuttlefish fishing, both registered during the first day of measurements (Table 2).

Furthermore, only four measurements during the two days of the campaign were made for the nape and chest exposure of two workers, respectively, FM 4 and FM 7. FM 4 nape exposure in the sea snail and cuttlefish fishing activity was between 166 to 380 J/m², while for FM 7, in the trawling fishing activity, chest exposure was found to be 98 and 69 J/m², respectively, during the first and the second day of the measurement campaign (Table 2 and Table 3).

Considering the UV doses registered by the dosimeter placed on a pair of sunglasses and worn by two investigators of the research team (M.B. and A.G.), we measured the ocular UV dose based on a three-hour monitoring period, and we registered 8 and 2 J/m², respectively, during the first and the second day of the measurement campaign.

## 4. Discussion

The results of the on-field individual UV exposure measurements in the group of fishermen indicate high UV exposure levels in North Italy, in particular in case of fishing activities carried out on boats with insufficient shading structures. The doses measured were quite variable during the two days of the campaign, considering the different boats and body areas monitored. Fishermen received daily 0.3 up to 5.4 standard erythemal doses (SED, where 1 SED = 100 J/m^2^ per day [6,29]), both values measured on the workers’ back. Overall, it has to be noted that even if the activities were monitored for few hours during partially clouded spring days, the suggested occupational exposure limit of 1–1.3 SED/day for skin exposure [32] was reached for 6 out of 14 individual daily measurements, also on boats with shading structures. Moreover, we monitored the fishermen only on the boats, but it has to be considered that they were exposed to the sunlight also when they came back to the port and unloaded the products collected to supply the local fish market.

Our results also confirm the relevant role of the working postures adopted in influencing the individual UV dose received by different body sites, such as the back, chest, and nape, in accordance with our previous findings in a group of fishermen from the Tyrrhenian Sea [15]. For practical reasons, as we could not interfere with the fishermen’s work activity, most of the dosimeters were placed on their back, and, overall, back exposure was found to be higher when compared with chest and nape exposure, with a single exception for a subject involved in trawling fishing during the first day of measurements. In this case, the fisherman had to stand upright for almost the entire monitored period to do his job, while usually, in most of the other working situations, the fishermen had to bent over to throw down and pick up the nets, and then to select the fish. In general, we found the highest UV exposures on the sea snail and cuttlefish fishing boat, which is less protected from direct sunlight: Fishermen engaged in this activity were exposed to UV on their back with a fraction between 14% and 65% of the cloud-modified erythemal UV dose coming from the sky in the same working period. Percentages of the ambient UV erythemal dose between 3% and 13% were estimated for the other fishing activities monitored, i.e., mussel fishing and trawling.

Our data, confirming intense UV exposures of the fishermen’s skin, together with scientific data showing high rates of precancerous skin lesions in maritime workers [24], indicate an urgent need to develop skin cancer preventive campaigns for fishermen, as sunlight exposure is a largely neglected occupational risk, at least in Europe [33,34]. It has to be noted that skin cancers are the most frequent neoplasms in Caucasians [34], and these data were recently confirmed in Italy [35]. Moreover, according to the CAREX study [36], solar radiation exposure in Italy, as well as in many other countries, is the occupational carcinogenic exposure involving the highest number of workers, just after passive tobacco smoke, e.g., in Italy, more than 700,000 outdoor workers, of whom 3200 are fishermen daily exposed to carcinogenic solar UV rays [36], and we should expect at least 1000 UV-inflicted occupational skin cancers (OSC) per year in Italy, but only a few dozen are reported each year to the compensation authority [37], and the same underreporting also happens in other countries [38,39]. Among the major issues in Europe for further improvements in the prevention of occupational solar UV exposure [7,40] there is the lack of established occupational exposure limits; only limits for artificial UV are available, but if we would apply these limits to outdoor work situations—and it can be possible, as solar UV radiation at the sea level is almost nonmeasurable below 300 nm of wavelength, so that the difference between the ICNIRP weighted and the erythemally weighted spectra can be considered negligible [6,14,27,28,29]—they would be largely exceeded, as shown in many studies [7,18,19,20,21,22].

Regarding fishermen’s eye exposure to solar UV, it has to be considered that albedo on the water is a relevant phenomenon, and the eye is not adequately protected from reflected UV rays. Moreover, fishing activities are often performed when the sun angle on the horizon is below 45°, with an increased possibility of direct ocular UV exposure [6,15,26]. To investigate ocular exposure on the water, we developed a special dosimeter to be worn on a pair of sunglasses: We registered an UV dose at the right side of the eye, which according to Coroneo’s effect also resembles the exposure of the medial part of the cornea and of the lens [41], varying from 2 to 8 J/m^2^ during the two days of the campaign. When comparing these exposures with the occupational exposure limits for the eye, i.e., 30 J/m^2^ [6,42], we found out that in only three hours, a percentage between 7% and 27% of the limit can be reached during fishing activities on the water (and again, it should be considered that the meteorological conditions were partially clouded and the measurements were performed in spring). This indicates an urgent need for the adoption of adequate eye protections in order to prevent the risk of adverse effects such as cataract, pterygium, and possibly macular degeneration [9,10,11]. In a previous study on fishermen from North Italy, cumulative cataract incidence on a period of eight years was present in 12.2% in the group of outdoor workers, with approximately a doubled risk when compared to the less exposed group [25].

A strength of our study is also the collection of available data on environmental erythemal UV dose from a closer meteorological station to reconstruct the percentages of individual vs. ambient UV exposure of the fishermen. This method may deserve further validations, as it may be useful in estimating UV risk in different outdoor work activities. As environmental UV data are widely and freely available, in case of a sufficient amount of measurements, the relations between individual vs. ambient UV exposure to be expected in different activities can be reconstructed, and this methodology can be easily applicable in various settings to evaluate occupational risk related to solar UV exposure. For these reasons, further studies with personal UV measurement campaigns are needed, to cover an adequate number of outdoor workers worldwide.

Our study also has some limitations, mainly related to the peculiarity of the work environment and the particular work organization, i.e., few fishermen working in small boats. First of all, we have to cite the relatively small number of person-days measured, with a monitoring period shorter than the standard 8 working hours, as many of the activities on the boats started before sunrise, with no UV exposure. Within the individual UV measurements collected, we found a quite large intervariability of results. These differences are not unexpected, and they depend on several factors, such as the relative position of the workers with respect to the sun in the different periods of the day according to the specific activity, the weather conditions of the month (May) when we performed the campaign, usually variable with different types of cloud cover and with quite large variability in the UV index during the two days of measurements, and also the type of working tasks on the different boats, which were not standardized. On the other hand, considering this specific work activity, it has to be noted that a good standardization of the exposure scenario is very difficult, or almost impossible. Another limitation is that on the small boats involved in the study, we were not able to find a stable and safe place where to put ambient dosimeters in an adequately representative position, and accordingly, we could not perform a full intercomparison of the measurements determined with the various individual dosimeters. In fact, we had to use different sensors, with different weighting functions (Table 1, Figure 1), to reach a sufficient number of fishermen with sensors placed at the same time on different body districts. For these reasons, we also performed a few measurements with non-erythemally weighted UV dosimeters, after an appropriate correction with normalization coefficients, calculated as explained in the Materials and Methods section. Furthermore, according to the manufacturer’s certificate, each dosimeter, properly calibrated compared to a reference source, has a specific relative uncertainty (according to the manufacturer, equal to 5% for UV-A sensors, 10% for unweighted UV-B sensors, and 12% for the erythemally and ICNIRP weighted dosimeters), and this uncertainty may even increase in the case of an “on field” UV measurement campaign, like the one we performed, representing a possible source of bias related to the UV exposure assessment of the study population. Moreover, uncertainty could be extremely relevant in some cases, e.g., when we would like to compare the results of the UV-A measurements with the ocular exposure limit. Regarding the body sites, our individual dosimeters were placed mainly on the back of the workers, so that we collected only few data on the possible exposure of other regions. Even though our electronic dosimeters were light, small, and easily portable, their application in a fixed position on the workers’ body raised some practical issues, and to standardize our measurements, we decided to collect most of the data from back exposure, which is certainly one of the fishermen’s most exposed body areas, if not adequately protected, as they are used to bending over to throw and collect the nets. To overcome this limitation, as a further development of this kind of on-field research, the use of mannequins, as widely reported [43,44,45], could be a good solution to standardize the study of exposure of different body areas according to working postures. Finally, again, in order to ensure no interference with normal work activity and, at the same time, to preserve a good quality of UV data, keeping the sensors in stable positions, avoiding wetting and other contaminations, we had to place the dosimeters on the clothes of the workers. However, according to the quite cold temperatures (as our campaign was performed during two quite windy and cloudy spring days), fishermen were wearing impermeable clothes, so that, in particular with the back dosimeters, we actually measured only a potential UV exposure of almost unexposed body areas. Only the nape and chest dosimeters were actually retrieving the exposure of uncovered body areas. When possible, the back sensors were also placed in the very upper part of the back, actually resembling with a good approximation the exposure of the uncovered nape. Nevertheless, we have to observe that, according to our previous investigation during the summer season [15], we found that, when the temperatures are higher, Italian fishermen are used to performing their activities wearing light fabric clothes, often with short sleeves, and sometimes, especially during the central hours of the day, they may decide to take off their shirts.

## 5. Conclusions

We found high individual UV exposure levels in a group of fishermen from North Italy, even if the measurement campaign was conducted during partially clouded spring days, with 43% of the daily personal UV measurements potentially exceeding the occupational limits, in case of exposure of uncovered skin areas. The highest exposures were collected for the sea snail and cuttlefish fishing activity. We also calculated the percentages of individual vs. environmental UV exposure, comparing our UV measurements with available cloud-modified erythemal UV dose data: The method elaborated can be considered promising for reliable estimates of UV risk in different work settings, once a sufficient amount of measurements is performed. UV monitoring campaigns and estimates of individual vs. environmental UV exposure can provide useful data to be communicated to the workers, in order to increase their risk perception and improve the adoption of adequate preventive measures and sun protections. UV risk should be considered in all outdoor occupations, and it is important to raise awareness on this almost neglected occupational threat, which is an extremely frequent cause of adverse health effects and occupational diseases. For these reasons, further implementation of appropriate preventive interventions and policies to protect outdoor workers are urgently needed.

## Figures and Tables

**Table 1:** Spectral responsivity of the electronic dosimeters used for the measurement campaign of individual UV exposure of the group of fishermen.

Dosimeter		Spectral Responsivity Range	Weighting Function	Measurement Range (W/m^2^)
UV-A	X2000	320–400 nm	unweighted *	0.02–3000
UV-B	X2000	280–320 nm	unweighted *	0.02–2500
UV-A ERYTHEMAL	X2000	320–400 nm	DIN 5050-1 2009 Erythemal actinic curve	0.002–30
UV-B ERYTHEMAL	X2000	250–320 nm	DIN 5050-1 2009 Erythemal actinic curve	0.03–5500
UV-A ICNIRP	X2000	320–400 nm	DIN EN 62471:2009	0.001–14
UV-B ICNIRP	X2000	280–320 nm	DIN EN 62471:2009	0.03–5000
UV-A	X2012	320–400 nm	CIE Erythemal action spectrum	0.002–30
UV-B/-C	X2012	250–320 nm	CIE Erythemal action spectrum	0.03–5500

* These instruments have a specific response, which is a function of the wavelengths.

**Table 2:** Individual solar UV exposure measured for the seven fishermen on the first day of measurements: 15th May. NB: Cloud-modified erythemal UV dose (*cme*UV*d*) = 2.6 kJ/m^2^.

Fishing Activity	Fisherman (FM)	Exposure Period Monitored (Minutes) and Corresponding Fraction of the *cme*UV*d*	Exposure on the Back (J/m²)	Exposure on the Chest/Nape (J/m²)	% of Personal vs. Ambient Exposure
Mussels Fishing	FM1	397′/1.71 kJ/m^2^	213	/	12.5
	FM2		79	/	4.6
	FM3		65	/	3.8
Sea Snails & Cuttlefish fishing	FM4	178′/0.83 kJ/m^2^	542	380 (nape)	65.3 (45.8 for nape)
	FM5		288	/	34.7
Trawling	FM6	261′/1.26 kJ/m^2^	84	/	6.7
	FM7		32	98 (chest)	2.5 (7.8 for chest)

**Table 3:** Individual solar UV exposure measured for the seven fishermen on the second day of measurements: 16th May. NB: Cloud-modified erythemal UV dose (*cme*UV*d*) = 3.5 kJ/m^2^.

Fishing Activity	Fisherman (FM)	Exposure Period Monitored (Minutes) and Corresponding Fraction of the *cme*UV*d*	Exposure on the Back (J/m²)	Exposure on the Chest/nape (J/m²)	% of Personal vs. Ambient Exposure
Mussels Fishing	FM1	210′/0.81 kJ/m^2^	29	/	3.6
	FM2		25	/	3.1
	FM3		71	/	8.8
Sea Snails & Cuttlefish fishing	FM4	180′/0.62 kJ/m^2^	284	166 (nape)	45.8 (26.8 for nape)
	FM5		84	/	13.5
Trawling	FM6	300’/1.56 kJ/m^2^	151	/	9.7
	FM7		129	69 (chest)	8.3 (4.4 for chest)
